# Association Between Antipsychotic Treatment and Neurological Adverse Events in Pediatric Patients: A Population-Based Cohort Study in Korea

**DOI:** 10.3389/fpsyt.2021.668704

**Published:** 2021-05-26

**Authors:** Soo Min Jeon, Susan Park, Soonhak Kwon, Jin-Won Kwon

**Affiliations:** ^1^BK21 FOUR Community-Based Intelligent Novel Drug Discovery Education Unit, College of Pharmacy, Research Institute of Pharmaceutical Sciences, Kyungpook National University, Daegu, South Korea; ^2^Department of Pediatric Neurology, Kyung-pook National University Children's Hospital, Kyungpook National University School of Medicine, Daegu, South Korea

**Keywords:** antipsychotic, pediatric, neurological adverse events, seizure, movement disorder, cohort study

## Abstract

**Background:** Potential adverse effects might be caused by increasing the number of antipsychotic prescriptions. However, the empirical evidence regarding pediatric psychiatric patients is insufficient. Therefore, we explored the antipsychotic-induced adverse effects focusing on the neurological system.

**Method:** Using the medical information of pediatric patients retrieved from the claims data of Health Insurance Review and Assessment in Korea, we identified those psychiatric patients who were started on antipsychotic treatment at age 2–18 years between 2010 and 2018 (*n* = 10,969). In this study, movement disorders and seizures were considered as major neurological adverse events. The extended Cox model with time-varying covariates was applied to explore the association between antipsychotic medication and adverse events.

**Findings:** Total 1,894 and 1,267 cases of movement disorders and seizures occurred in 32,046 and 33,280 person-years, respectively. The hazard risks of neurological adverse events were 3–8 times higher in the exposed to antipsychotics period than in the non-exposure period. Among the exposure periods, the most dangerous period was within 30 days of cumulative exposure. High doses or polypharmacy of antipsychotics was associated with increased risks of neurological adverse events. Among individual antipsychotics, haloperidol showed the highest risk of developing movement disorders among the examined agents. Quetiapine showed a lower risk of developing movement disorders but a higher risk of developing seizures than risperidone.

**Conclusion:** These findings suggest that antipsychotics should be used with caution in pediatric patients, especially regarding initial exposure, high dose, and polypharmacy.

## Introduction

Recently, antipsychotic medications are prescribed more frequently for pediatric patients due to the increasing availability of antipsychotic drugs and the expansion of their indications (1–4). This increased use of antipsychotics has raised concerns about their potential adverse effects. For example, movement disorders, one of neurological adverse events, are much frequently occurring adverse events associated with antipsychotic treatments (5–8). Several studies have reported that antipsychotics treatment could provoke the development of movement disorders such as tardive dyskinesia and extrapyramidal symptoms (5, 9, 10), and which risk with second-generation antipsychotics was much lower compared with first-generation antipsychotics. However, one previous systematic review and meta-analysis of randomized controlled trials in children and adolescents revealed that risperidone and aripiprazole had a higher rate of extrapyramidal symptoms in placebo groups (7). Seizures can be other hidden neurological adverse events caused by antipsychotic treatments because antipsychotics can reduce the seizure threshold (5, 11).

Antipsychotics treatment patterns, such as exposure time and drug dose, could be important factors for neurological adverse events. Previous studies on the neurological adverse events of antipsychotics have evaluated the use of antipsychotics at a specific time point or within a limited time period (12–14). However, physicians may change drug dosage or switch between antipsychotic agents at any time depending on the clinical response, which may affect the changes in the risk of antipsychotics. This might lead to under- or overestimation of the risk of antipsychotics on neurological adverse events in the analysis.

Child and adolescents tend to experience more prevalent and severe side effects of antipsychotics compared with adults (15, 16). Furthermore, a substantial number of pediatric patients are concomitantly treated with >2 antipsychotic drugs (17). This treatment pattern, also known as antipsychotic polypharmacy, may increase the risk of side effects compared with treatment with one antipsychotic medication (18), because this pattern tends to induce increasing the drug dose and the potential drug–drug interaction (19–21). However, the evidence regarding the safety of antipsychotic polypharmacy is insufficient, particularly for pediatrics.

Therefore, this study investigated the risks of developing movement disorders and seizures for pediatric patients according to the patterns of antipsychotic treatments, including the exposure duration, drug dose, and polypharmacy. We used a cohort design with a time-dependent method to track the changes in antipsychotic treatments over time. The findings of this study could be used to develop strategies for a safe antipsychotic therapy in the pediatric population with psychiatric disorders.

## Method

### Data Source

A population-based, retrospective cohort study of pediatric patients was conducted using data collected from the Health Insurance Review and Assessment (HIRA) database recorded from 2008 to 2018. The entire Korean population (~50 million people) is under the universal health coverage system, including the National Health Insurance Service (NHIS) (97%) or the medical aid program (3%). The HIRA database is constructed using the record of claims data collected in the process of reimbursing claims for healthcare providers; therefore, this database stores medical information for almost the entire national population. From this database, we used the anonymized personal code, age, sex, diagnosis, prescription records, and sociodemographic records for our analysis. The diagnosis of disease was recorded as per the International Classification of Diseases, 10th Edition (ICD-10 code). The prescription records provided information about active ingredients, issue dates, treatment duration, and dose and route of administration. HIRA provides research data after anonymizing the patient's identification information. Therefore, we could not obtain the patient's personal information in the data. The Institutional Review Board (IRB) of Kyungpook National University (IRB number: KNU 2018-0141) waived the patient/guardian consent requirement and approved this study.

### Study Population

For this retrospective cohort study, we defined inclusion criteria as (1) psychiatric patients, (2) age 2–18 years, (3) new prescriptions of antipsychotics to treat mental illness. Exclusion criteria were considered as (1) medical history of movement disorder or seizures before the outcome follow-up, (2) prescription of injectable antipsychotics, and (3) over 18 years of age during the follow-up period.

Detailed descriptions of the process of creating the eligible cohort were as follows. Firstly, we identified the patients who prescribed antipsychotic medications at age 2–18 years from January 1, 2008, to December 31, 2018. Then, to clarify new users of antipsychotics treatment, patients who initiated antipsychotics treatment in the first 2 years (2008/2009) were excluded (n = 10,100). Patients who used injectable antipsychotic at the first date of antipsychotic prescription were excluded to reduce the heterogeneity of disease severity (*n* = 1,507) (22). We also excluded the patients if they had no history of psychiatric diagnosis (ICD-10 code, F) for 1 year before the index date to exclude antipsychotics prescriptions for non-psychiatric indications (*n* = 142).

**Figure 1:**
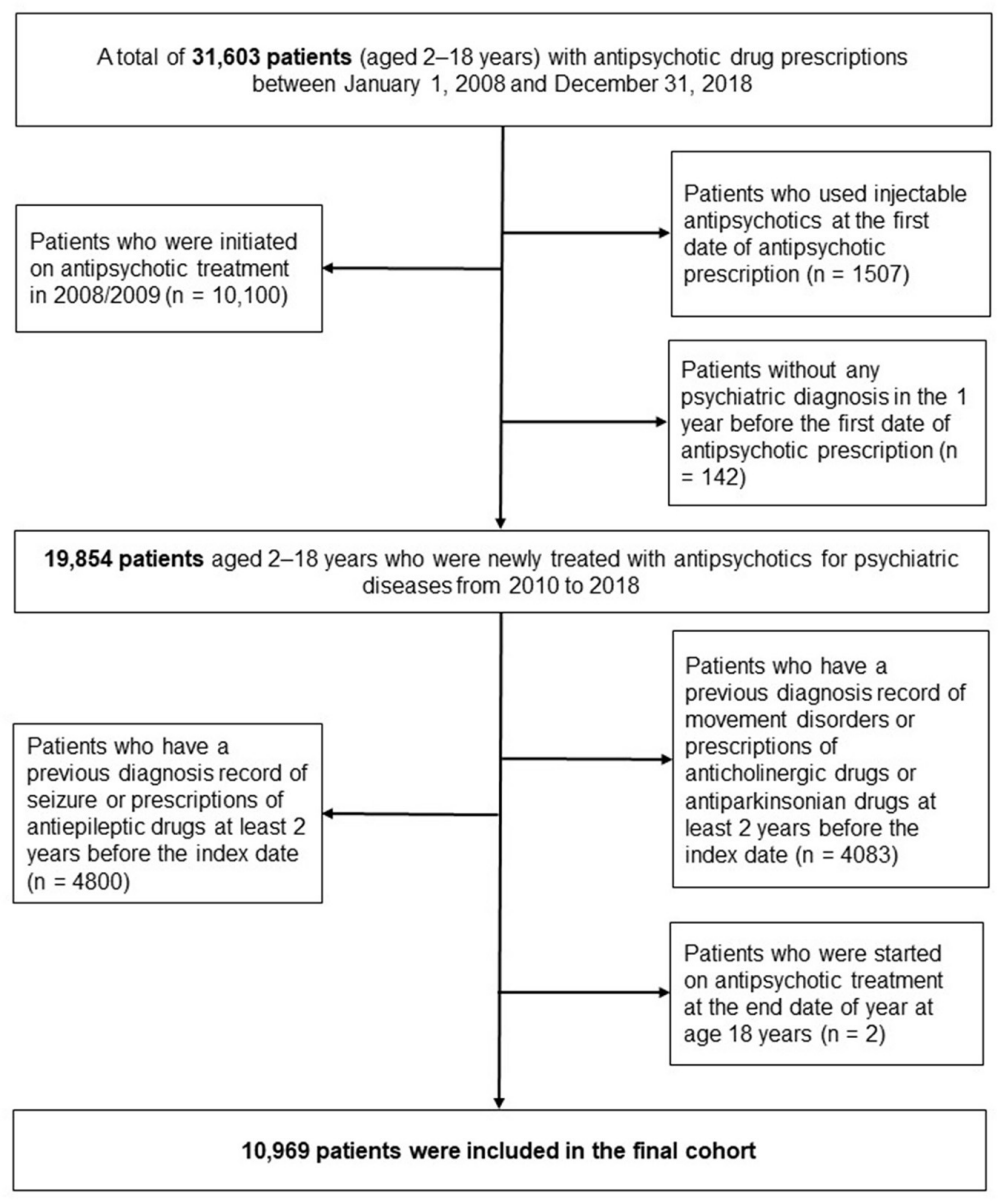
Flow chart for identifying study patients newly initiated on antipsychotic treatment at age 2–18 years.

Of them, to assess the incidence of neurological adverse events, we excluded patients if they had any history of movement disorders and seizures in the claims database before the index date. In this process, we excluded (1) 4,083 patients diagnosed with movement disorders or used anticholinergics/antiparkinsonians and (2) 4,800 patients diagnosed with seizures or used antiepileptics. The detailed codes of diagnosis and medications are presented in Supplementary Tables 1–3. Next, we excluded the patients who had index date at the end date of the year at age 18. Finally, 10,969 pediatric patients were selected for this analysis (Figure 1).

### Neurological Adverse Events

The incidence of movement disorders and seizures was evaluated as representative diseases among various neurological adverse events, and they were evaluated separately in the analyses. Therefore, in our study, we constructed two cohorts with different follow-up durations for each outcome. The detection of movement disorders was based on ICD-10 diagnosis codes as follows: G20, G21, G21.1, G21.2, G21.8, G21.9, G24, G24.0, G24.2, G25, G25.0, G25.1, G25.4, G25.6, and G25.9. Seizure events were defined as cases with antiepileptic prescription or an electroencephalographic (EEG) examination within 30 days after the diagnosis of “Other and unspecified convulsions” (ICD-10 code R56.8) or Epilepsy (ICD-10 code G40).

### Antipsychotic Exposure

We included both typical and atypical antipsychotic medications defined by the Anatomical Therapeutic Chemical classification code N05A, except lithium. For the majority of the patients, the antipsychotic treatment regimen could change during the follow-up period. Because the antipsychotic prescription is not constant over time, particularly in pediatric patients, the exposure status of antipsychotics was measured as time-varying variables. The person-time during the follow-up period was categorized into “exposure period” and “non-exposure period,” based on the supplying days of the antipsychotic prescription. Each person-time was defined as the “exposure period” at the time point of prescribing the antipsychotics and continued until 14 days after the end date of the prescription. All remaining person-times were defined as the “non-exposre period.”

For detailed analyses of adverse effects according to the antipsychotic use, we subdivided the exposure period as follows: cumulative duration of use (months) and antipsychotic polypharmacy (monotherapy and polypharmacy). The cumulative duration of antipsychotic use was measured as the sum of total days with antipsychotic prescription from the index date. Monotherapy and polypharmacy were determined by the number of antipsychotic agents prescribed on the same day. The time when two or more antipsychotic agents were prescribed on the same day was defined as polypharmacy period. For a detailed risk analysis for individual antipsychotic agent, we subdivided the monotherapy period based on the each prescribed agent as follows: risperidone, aripiprazole, quetiapine, olanzapine, and others.

The average daily dose of antipsychotic agents was also measured during the exposure period to adjust for the antipsychotic dose in the analysis of polypharmacy. The average daily dose was calculated by dividing the sum of total prescribed dosage during each exposure period by the total number of observation days, which was converted into chlorpromazine equivalents for comparative evaluation (23). Next, they were categorized into four groups based on the quartiles of average daily dose by age group at each prescription date.

### Covariates

To control for other possible confounders associated with neurological adverse events, we included various covariates as follows: age, sex, insurance type, psychiatric hospitalization, mental health conditions, and concomitant use of other psychotropic medications. Among these covariates, age, psychiatric hospitalization, and other psychotropic medications were measured in a time-dependent manner to identify the changing conditions. We considered mental health conditions in Supplementary Table 4. Other psychotropic medications were considered as anticholinergic drugs, antiepileptic drugs, antianxiety drugs, SSRIs/SNRIs, tricyclic antidepressants, MAO inhibitors, methylphenidate, non-stimulant drugs for attention deficit hyperactivity disorder (ADHD), and lithium (Supplementary Table 2). However, anticholinergic and antiepileptic medications were excluded from the covariates in the analysis of movement disorders and seizures, respectively, because some psychotropic drugs were used to define the outcome occurrence.

### Statistical Analysis

Personal characteristics of the patients at baseline were presented as numbers and proportions. Crude incidence rates of outcome diseases were evaluated as the number of new events per 100 person-years during the follow-up period. An extended Cox model was fitted wherein the antipsychotic prescription was treated as a time-dependent predictor. This method is widely used in medical literature to consider the violations of proportional hazards (i.e., value changes over time) (24, 25). Two separate multivariable extended Cox models were constructed, one for movement disorders and the other for seizures. First, we evaluated the risk of each outcome during the exposure period compared with the non-exposure period. Second, restricted to the exposure period, we assessed the effects of cumulative exposure duration, polypharmacy, and the individual antipsychotic agent on the outcome diseases. In these two steps, the models were commonly adjusted for age, sex, insurance type, psychiatric diagnosis, hospitalizations, and other psychiatric medications. Third, we conducted additional analyses on the effect of antipsychotic polypharmacy on the outcome diseases after controlling for time-dependent quartiles of the average daily dose. The risk of movement disorders or seizure was presented as Hazard ratios (HR) with 95% Confidence intervals (CIs).

To evaluate the uncertainty of definition of exposure period, sensitivity analyses were performed on the changes in the carry-over effect period. We also restricted the follow-up period to the first year of initiation of treatment to minimize the effect of variation of psychiatric diagnosis from baseline.

All statistical analyses were conducted using SAS Enterprise Guide 6.1 (SAS Institute Inc., Cary, NC, USA).

### Role of the Funding Source

The funder of this study had no role in the study design, data collection, data analysis, data interpretation, or writing of the report and had no access to the raw data. The corresponding author had full access to all the data and had the final responsibility of the decision to submit for publication.

## Results

Table 1 shows the demographic characteristics and psychiatric diagnoses of the patients. Of 10,969 patients, more than half (61.27%) were men and aged between 13 and 18 years (66.67%). A majority of the study population had health insurance (88.29%) at baseline. The most frequently prescribed antipsychotic drugs at the index date were risperidone (45.98%), followed by aripiprazole (31.06%). For 1 year before the initiation of antipsychotic treatment, the majority of study patients were diagnosed with depression (41.77%), followed by ADHD (33.10%) and schizophrenia spectrum (29.58%).

**Table 1:** Baseline demographics of study subjects who were initiated on antipsychotic treatment, 2010–2018 (*n* = 10,969).

**Characteristic**	***n***	**%**
**Sex**		
Male	6,721	61.27
Female	4,248	38.73
**Age at cohort entry**		
Mean (SD)	13.42 (3.75)	
2–6	547	4.99
7–12	3,109	28.34
13–18	7,313	66.67
**Insurance type**		
Health insurance	9,684	88.29
Medical aid	1,285	11.71
**Prescribing antipsychotic drugs at cohort entry**		
Risperidone	5,043	45.98
Aripiprazole	3,407	31.06
Quetiapine	641	5.84
Haloperidol	370	3.37
Olanzapine	203	1.85
Other monotherapy	867	7.90
Polypharmacy	438	3.99
**Mental health conditions for one year before the index date**		
Anxiety disorder	2,462	22.45
Depression	4,582	41.77
ADHD	3,631	33.10
Mental retardation	1,178	10.74
Tic disorder	1,373	12.52
Bipolar disorder	1,266	11.54
Schizophrenia spectrum	3,245	29.58
Autism spectrum disorder	986	8.99

*SD, standard deviation; ADHD, attention deficit hyperactivity disorder*.

Over a mean follow-up period of 2.02 years (standard deviation = 2.28), generating 32,045 person-years for movement disorders, there were 1,894 individuals who experienced movement disorders. The number of seizures during a mean period of 3.03 years was 1,267. Table 2 shows the incidence rates and hazard ratios for two neurological adverse events. The incidence rate of movement disorders was higher in the antipsychotic exposure period than in the non-exposure period (exposure period 12.29 cases/100 person-years; non-exposure period 1.80 cases/100 person-years). After adjustment for covariates, the exposure period was associated with 8.17-fold increased risk for movement disorder events (HR = 8.17; 95% CI 7.16–9.33) compared with the non-exposure period. Likewise, seizures occurred more frequently during the antipsychotic exposure period compared with during the non-exposure period (exposure period 1.76 cases/100 person-years; non-exposure period 6.66 cases/100 person-years). The adjusted HRs for seizure incidence were significantly greater during the exposure period than during the non-exposure period (HR = 3.47; 95% CI 2.99–4.03).

**Table 2:** Risk of developing movement disorders or seizures according to the exposure status of antipsychotics.

**Exposure status**	**Cases**	**Person-years**	**Incident rates per 100 person-years**	**Adjusted HR (95% CI)^a^**
**Movement disorders**
Non-exposure period	350	19,478	1.80	1.00 (reference)
Exposure period	1,544	12,568	12.29	8.17 (7.16–9.33)
Exposure status	Cases	Person-years	Incident rates per 100 person-years	Adjusted HR (95% CI)^b^
**Seizures**
Non-exposure period	342	19,384	1.76	1.00 (reference)
Exposure period	925	13,896	6.66	3.47 (2.99–4.03)

a *Adjusted for sex, age, insurance type, inpatient history, and psychiatric diagnosis. To consider the severity of psychiatric disorder related to seizure occurrence, covariates of inpatient history and other psychiatric medication use considered in a time-dependent manner were used in these models. Other psychiatric medication was included as antianxiety drugs, antidepressant drugs, stimulants for ADHD, non-stimulants for ADHD, antiepileptic drugs, and lithium*.

b *Adjusted for sex, age, insurance type, inpatient history, and psychiatric diagnosis. To consider the severity of psychiatric disorder related to seizure occurrence, covariates of inpatient history and other psychiatric medication use considered in a time-dependent manner were used in these models. Other psychiatric medication was included as anticholinergic drugs, antianxiety drugs, antidepressant drugs, stimulants for ADHD, non-stimulants for ADHD, and lithium*.

*HR, hazard ratio; CI, confidence intervals; ADHD, attention deficit hyperactivity disorder*.

**Figure 2:**
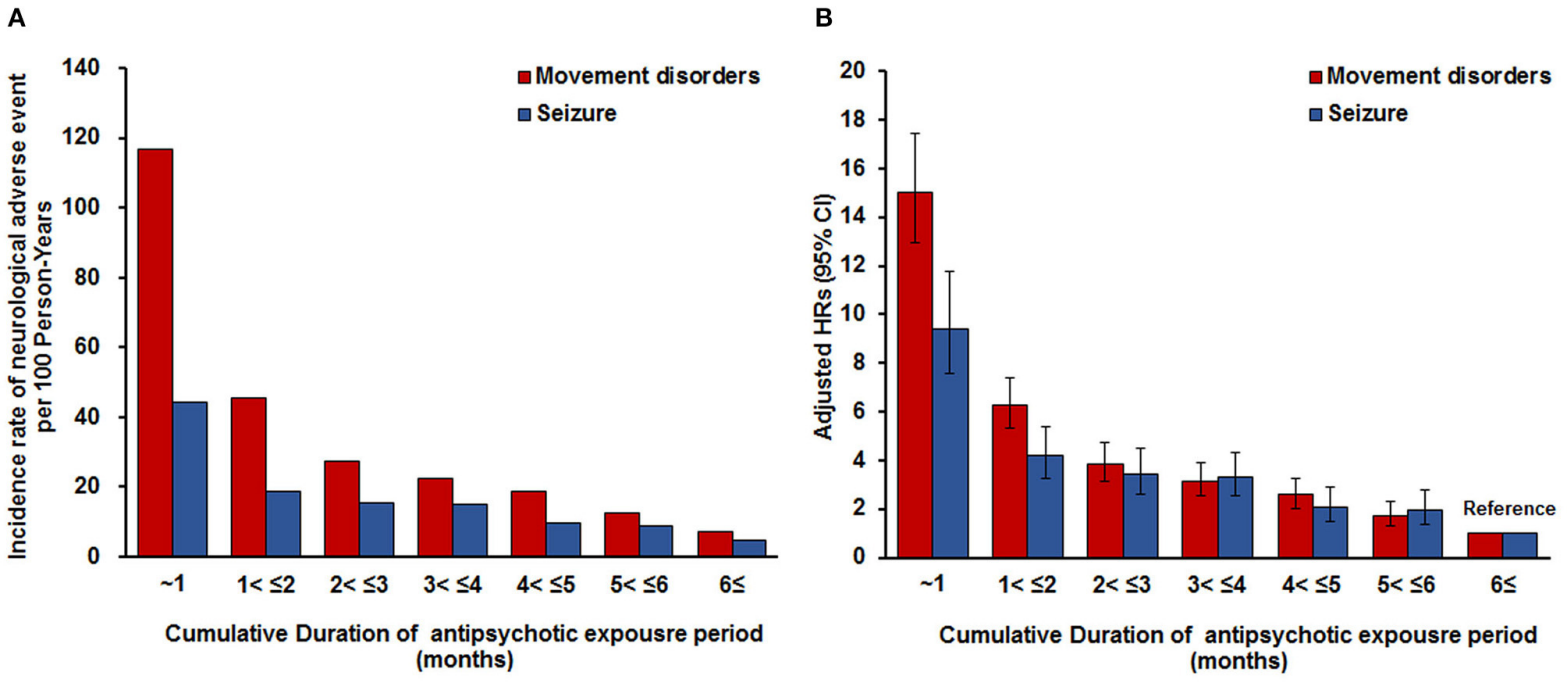
Incidence rate of movement disorders or seizures per 100 person-years according to the cumulative duration of antipsychotic exposure period. **(A)** Adjusted for sex, age, insurance type, inpatient history, and psychiatric diagnosis. To consider the severity of psychiatric disorder related to seizure occurrence, covariates of inpatient history and other psychiatric medication use considered in a time-dependent manner were used in these models. Other psychiatric medication was included as antianxiety drugs, antidepressant drugs, stimulants for ADHD, non-stimulants for ADHD, antiepileptic drugs, and lithium. **(B)** Adjusted for sex, age, insurance type, inpatient history, and psychiatric diagnosis. To consider the severity of psychiatric disorder related to seizure occurrence, covariates of inpatient history and other psychiatric medication use considered in a time-dependent manner were used in these models. Other psychiatric medication was included as anticholinergic drugs, antianxiety drugs, antidepressant drugs, stimulants for ADHD, non-stimulants for ADHD, and lithium. ADHD, attention deficit hyperactivity disorder.

Analysis of the cumulative duration of antipsychotic exposure revealed higher risks for movement disorders and seizures in groups with a shorter duration of antipsychotic treatment (Figure 2). The period with <1 month of cumulative duration of exposure to antipsychotics had 15.01-and 9.44-fold higher risks for movement disorders and seizures, respectively, than the period with exposure for >6 months (Movement disorders: HR = 15.01, 95% CI 12.93–17.44; Seizures: HR = 9.44; 95% CI 7.56–11.78).

Table 3 presents the analysis results of the risk of developing movement disorders or seizures according to antipsychotic polypharmacy. Antipsychotic polypharmacy was found to be independently associated with an increased risk of developing movement disorders compared with monotherapy, irrespective of controlling for drug dose (Model 1: HR = 1.76, 95% CI 1.55–2.00; Model 2: HR = 1.44, 95% CI 1.26–1.65). Furthermore, the risk of developing movement disorders was elevated with increasing average daily dose of antipsychotics (moderate dose HR = 1.28, 95% CI 1.06–1.55; high-dose HR = 1.63, 95% CI 1.37–1.94; very high-dose HR = 1.99, 95% CI 1.65–2.39). For seizures, the antipsychotic polypharmacy was associated with increased seizure risk in the analysis without adjusting for drug dose (Model 1 HR = 1.33, 95% CI 1.12–1.58), whereas no significant difference was observed in the seizure risk between monotherapy and polypharmacy after adjusting for the antipsychotic dose (Model 2 HR = 1.17, 95% CI 0.98–1.39). The magnitude of seizure risk exhibited a linear relationship with increasing antipsychotic dose (moderate dose HR = 1.16, 95% CI 0.93–1.44; high-dose HR = 1.26, 95%, CI 1.03–1.55; very high-dose HR = 1.61, 95% CI 1.29–2.02).

**Table 3:** Risk of developing movement disorders or seizures according to antipsychotic polypharmacy during the exposure period of antipsychotics.

**Treatment regimen and dose of antipsychotics**	**Cases**	**Person-years**	**Incidence rate per 100 person-years**	**Adjusted HR^a^ (95% CI)**	
				**Model 1**	**Model 2**
**Movement disorders**					
Monotherapy	1,071	10,206	10.49	1.00 (Reference)	1.00 (Reference)
Polypharmacy	473	2,362	20.03	1.76 (1.55–2.00)	1.44 (1.26–1.65)
Dose^b^					
Low dose	211	32,723	6.45	–	1.00 (Reference)
Moderate dose	287	36,164	7.94	–	1.28 (1.06–1.55)
High dose	495	44,497	11.12	–	1.63 (1.37–1.94)
Very high dose	551	37,428	14.72	–	1.99 (1.65–2.39)
**Treatment regimen and dose of antipsychotics**	**Cases**	**Person-years**	**Incidence rate per 100 person-years**	**Adjusted HR^c^ (95% CI)**	
				**Model 1**	**Model 2**
**Seizures**					
Monotherapy	643	10,469	6.14	1.00 (Reference)	1.00 (Reference)
Polypharmacy	282	3,427	8.23	1.33 (1.12–1.58)	1.17 (0.98–1.39)
Dose^b^					
Low dose	147	2,815	5.22	–	1.00 (Reference)
Moderate dose	196	3,306	5.93	–	1.16 (0.93–1.44)
High dose	269	4,032	6.67	–	1.26 (1.03–1.55)
Very high dose	313	3,743	8.36	–	1.61 (1.29–2.02)

a *Adjusted for sex, age, insurance type, inpatient history, and psychiatric diagnosis. To consider the severity of psychiatric disorder related to seizure occurrence, covariates of inpatient history and other psychiatric medication use considered in a time-dependent manner were used in these models. Other psychiatric medication was included as antianxiety drugs, antidepressant drugs, stimulants for ADHD, non-stimulants for ADHD, antiepileptic drugs, and lithium*.

b *Average daily dose was calculated as chlorpromazine equivalent dose and categorized into four groups according to time-varying age in each cohort*.

c *Adjusted for sex, age, insurance type, inpatient history, and psychiatric diagnosis. To consider the severity of psychiatric disorder related to seizure occurrence, covariates of inpatient history and other psychiatric medication use considered in a time-dependent manner were used in these models. Other psychiatric medication was included as anticholinergic drugs, antianxiety drugs, antidepressant drugs, stimulants for ADHD, non-stimulants for ADHD, and lithium*.

*HR, Hazard ratio; CI, Confidence intervals; ADHD, attention deficit hyperactivity disorder*.

**Figure 3:**
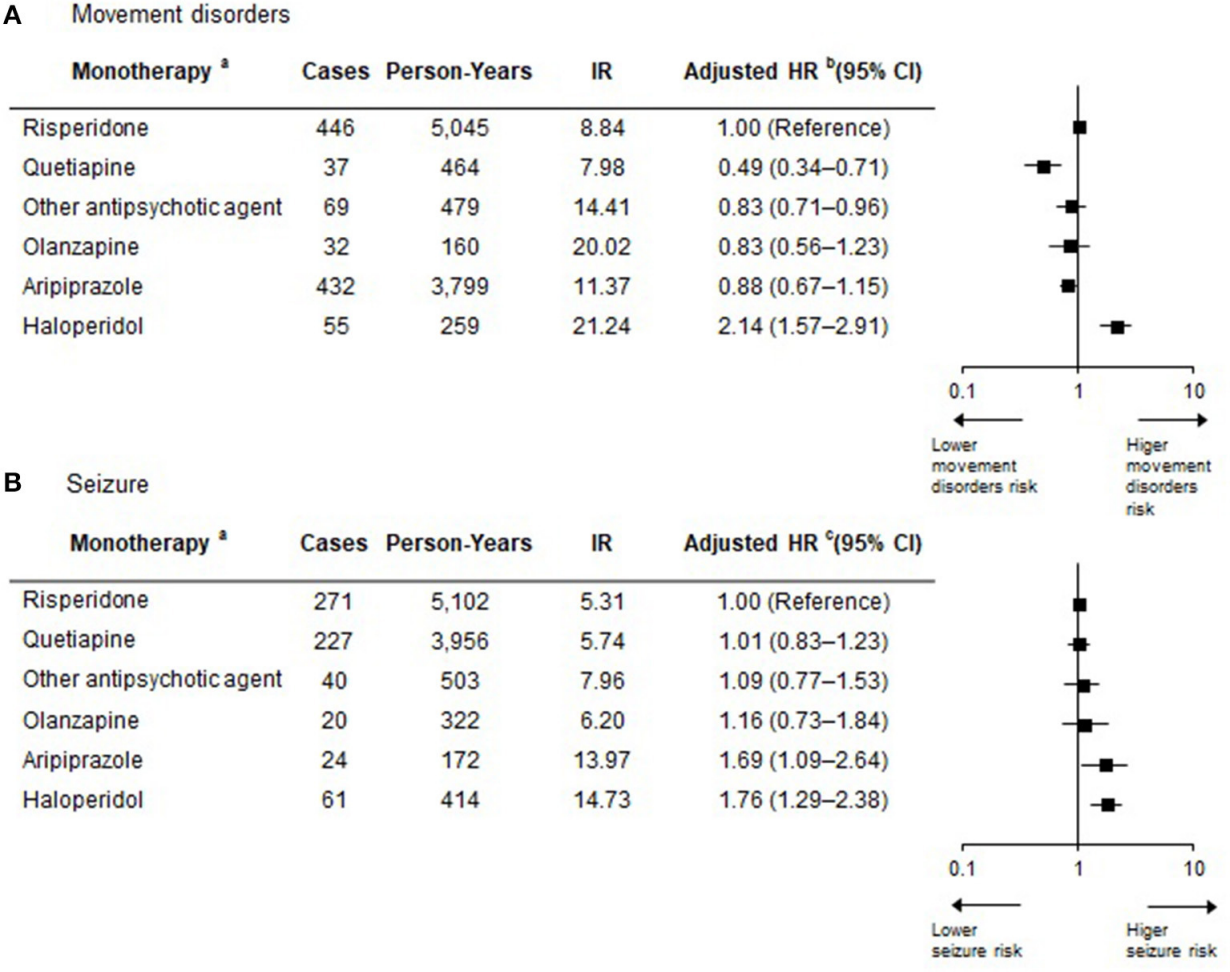
Risk of developing movement disorders or seizures according to antipsychotic agent during the antipsychotic exposure period. (a) Antipsychotic exposure period with polypharmacy is not displayed in the figure, but it is included in the extended Cox regression model to allow for appropriate estimation of treatment effects. (b) Adjusted for sex, age, insurance type, inpatient history, and psychiatric diagnosis. To consider the severity of psychiatric disorder related to seizure occurrence, covariates of inpatient history and other psychiatric medication use considered in a time-dependent manner were used in these models. Other psychiatric medication was included as antianxiety drugs, antidepressant drugs, stimulants for ADHD, non-stimulants for ADHD, and lithium. (c) Adjusted for sex, age, insurance type, inpatient history, and psychiatric diagnosis. To consider the severity of psychiatric disorder related to seizure occurrence, covariates of inpatient history and other psychiatric medication use considered in a time-dependent manner were used in these models. Other psychiatric medication was included as anticholinergic drugs, antianxiety drugs, antidepressant drugs, stimulants for ADHD, non-stimulants for ADHD, and lithium. IR, Incidence rate per 100 person-years; HR, Hazard ratio; CI, Confidence intervals; ADHD, attention deficit hyperactivity disorder.

We also found that the risk of developing movement disorders or seizures was different according to individual antipsychotic agent (Figure 3). Haloperidol was associated with an increased risk of developing movement disorders compared with risperidone (HR = 2.14; 95% CI 1.57–2.91), but no significant difference was detected in seizure development between these agents. For quetiapine, the lowest risk of developing movement disorders was observed among the individual antipsychotic agents, which was ~49% lower than that of risperidone (HR = 0.49; 95% CI 0.34–0.71), but this agent had the highest risk of developing seizures (HR = 1.76, 95% CI 1.29–2.38). Regarding seizures, olanzapine had a higher seizure risk (HR = 1.69, 95% CI 1.09–2.64) than risperidone, but the risk of developing movement disorders was similar to that of risperidone. Aripiprazole showed no significant difference in the risk of developing both movement disorders and seizures compared with risperidone.

In the sensitivity analysis with 7 days of carry-over effect period, we confirmed the associations between antipsychotic use and neurological adverse events similar to those in our primary analysis (Supplementary Tables 6–9). We also confirmed that antipsychotic polypharmacy was associated with increased risk of developing movement disorders when restricted to a follow-up period of 1 year, whereas seizure risk was not elevated in polypharmacy compared with monotherapy (Supplementary Tables 10–12).

## Discussion

In this cohort study of children and adolescents who were newly treated with antipsychotic agents, we demonstrated the presence of associations between antipsychotic treatment and incidences of movement disorders and seizures. Moreover, during the antipsychotic exposure period, the incidence rates of movement disorders and seizures were found to be the highest in the cumulative duration of 1–30 days, which were gradually decreased with increased duration of exposure. For movement disorders, antipsychotic polypharmacy was identified as a significant risk factor for movement disorders, irrespective of the antipsychotic drug dose. However, antipsychotic polypharmacy was not associated with seizure risk after adjustment for the drug dose. Among the individual antipsychotic agents, haloperidol exhibited the highest risk of developing movement disorders, and quetiapine was associated with a 49% lower and a 76% higher risk of developing movement disorders and seizures, respectively, compared with risperidone.

Using the extended Cox model with time-varying covariates, we found that antipsychotic polypharmacy was associated with increased risks of developing movement disorders and seizures compared with monotherapy. However, these risks were decreased or disappeared in the analyses after adjusting for the drug dose. These findings suggest that the increased risk of adverse events associated with antipsychotic polypharmacy was affected by the drug dosage. Previous studies have demonstrated that a correlation exists between antipsychotic polypharmacy and high dose (19–21), and the average daily dose of polypharmacy was approximately six times higher than monotherapy in our data (Supplementary Table 13). Furthermore, it could be affected by the blood concentration of the antipsychotic agent induced by potential drug–drug interaction. Each antipsychotic agent has a distinctive metabolism profile involving cytochrome P450, but these agents could also undergo potential drug–drug interactions by inhibiting or inducing a specific enzyme responsible for the agent's metabolism (26). For instance, *in vitro* data have reported that clozapine inhibits CYP2C9, CYP2C19, CYP2D6, and CYP3A (27), which are involved in the metabolism of other antipsychotic agents. Therefore, drug interactions can lead to unexpected high plasma levels of antipsychotics (11), which probably cause a greater incidence of movement disorders.

We also confirmed that antipsychotic treatments were dose-dependently associated with movement disorders and seizures in pediatric patients, which is consistent with previous studies on adults (28–30). Although atypical antipsychotic agents have been recognized to have lower risk of developing movement disorders than typical antipsychotic agents, high-dose atypical agents have a similar risk of developing movement disorders as that of low-dose typical antipsychotic agents (30). Among atypical antipsychotic agents, risperidone showed a clear dose–response relationship with the risk of developing movement disorders (31), and olanzapine and ziprasidone were associated with risk of developing movement disorders (particularly akathisia) at higher doses (32). Likewise, the dose effect of antipsychotics on seizure incidence has been reported previously (33–37). For instance, clozapine exhibited a clear dose–response relationship in the increased risk of developing seizures. The incidence rate of seizures in patients using clozapine was ~1% when administered <300 mg/day, ~2.5% when administered 300–599 mg/day, and ~4.4% when administered >600 mg/day (34). Even with other antipsychotic agents, several seizure cases have been reported in patients receiving high-dose antipsychotic therapy (33, 35–37). Therefore, the American Psychiatric Association current clinical guideline recommend a low starting dose and slow dosage escalation to minimize the risk of developing seizures (38). In our study, the incidence rates of neurological adverse events were the highest when exposed to antipsychotic drugs for <1 month. Several previous studies have reported that the majority of movement disorders associated with antipsychotic use developed within few weeks after the initiation of treatment (39–41). Another previous study reported that the association between antipsychotic use and parkinsonism was more intensified in recently treated patients (28). Our previous study had also demonstrated that the current use of antipsychotics (within 90 days before the incidence date of seizures) had a greater risk of developing seizures than that of consistent use (>90 days before the incidence date of seizures) (12).

Typical antipsychotic agents, including haloperidol, have a stronger affinity to dopaminergic D2 receptors than atypical antipsychotic agents (30, 42); therefore, they are well-known to have a higher risk of developing movement disorders than atypical drugs. Consistent with previous studies (43–46), our study results disclosed a higher risk of developing movement disorders in pediatric patients undergoing haloperidol treatment compared with those receiving risperidone treatment. Within atypical antipsychotics, individual antipsychotics have different sizes of strength of blockade for D2 receptors. For instance, quetiapine and risperidone were found to have low and high affinities to the antagonistic D2 effect, respectively (42, 43). In our analysis, quetiapine showed a lower risk of developing movement disorders than risperidone, and which is in line with previous studies that compared the neurological adverse effect risk profile for several second-antipsychotics in children and adolescents (47, 48). However, as all antipsychotic agents have some degree of D2 receptor affinity (42), there are no antipsychotic agents free from the risk of developing movement disorders. The incidence rate of movement disorders during the exposure period of all antipsychotic agents was much higher than that during the non-exposure period in our results. In the case of seizures, olanzapine and quetiapine showed a higher seizure risk than risperidone in our analysis, although controversy still exists that risperidone carries a lower risk than olanzapine and quetiapine (49). Olanzapine was generally known to be associated with a higher seizure incidence and a greater risk of EEG abnormalities than other antipsychotic agents (50–52). Although quetiapine has been less reported in the context of high seizure risk than olanzapine, some pharmacovigilance studies (52, 53) and case reports (33, 37, 54, 55) have also reported its association with seizure occurrence. Both these agents are closely related structurally to clozapine, which could explain the relatively higher seizure risk than other antipsychotic agents (53).

### Strengths

This is the first population-based new-user cohort study to explore the adverse effects of antipsychotic treatment in pediatric patients, considering the time-varying antipsychotic exposure. Restricting the cohort to new users could be the best method to reduce bias of unmeasured confounding that may alter medication use (56). Moreover, the extended Cox model was applied to appropriately evaluate the exposure status of antipsychotics that varied over time. This method has the advantage of reducing the immortal time bias (57) and clearly explains the attribution of exposure at the time of occurrence event.

### Limitations

There were some possible limitations in this study. First of all, due to the inherent limitation of study based on claims database, the definition of our outcomes could not be verified with clinical information. Furthermore, the exposure status of medications might potentially be misclassified because we could not measure the actual medical adherence to the prescribed medications. Considering the poor adherence of psychotropic medication in patients with mental health disorder (58), the measurement of antipsychotics exposure might be overestimated. Second, we could not completely control all confounders associated with the occurrence of neurological adverse events in an observational study. To compensate for this limitation, we used several time-invariant and time-varying covariates that we could possibly measure. We also performed a sensitivity analysis restricted to the follow-up period until 1 year to reduce the bias of time-invariant covariates. Third, due to lack of information, it was impossible to adjust the possible confounding effect of substance use on the occurrence of movement disorders and seizures. However, considering the lower prevalence (<1%) of illegal drug use in Korean adolescents (59), the effect of substance use on our results would be limited.

## Conclusion

In this large population-based cohort study, we found that pediatric patients faced an increased risk of developing movement disorders or seizures upon receiving antipsychotics, which was intensified with initial exposure, high dose, and polypharmacy. We also observed that the increased risks for neurological adverse events when prescribed polypharmacy were mediated by the antipsychotic dose. For the individual antipsychotic agents, each exhibited a different tendency of developing movement disorders and seizures. These findings underscored the need for carefully weighing the benefits and potential adverse effects when prescribing antipsychotics for pediatric patients with psychiatric diseases.

## Data Availability Statement

The data analyzed in this study is subject to the following licenses/restrictions: this study used Health Insurance Review and Assessment (HIRA) database. HIRA forbids the transfer, rent, or sale of the database to any third party other than the researcher, who obtained the approval for the provided database. Requests to access these datasets should be directed to HIRA; Official website of HIRA: https://opendata.hira.or.kr; Contact information of data access committee: +82-33-739-1083.

## Author Contributions

J-WK had full access to all the data in the study and takes the responsibility of integrity of the data and accuracy of the data analysis. SJ, SP, and J-WK were responsible for the study concept, design, and acquisition. SJ was involved in statistical analysis. SJ and SP drafted the manuscript. All authors were involved in the interpretation of data and critically revised the manuscript for important intellectual content.

## Conflict of Interest

The authors declare that the research was conducted in the absence of any commercial or financial relationships that could be construed as a potential conflict of interest.
