# Supplementary material for: Association Between Antipsychotic Treatment and Neurological Adverse Events in Pediatric Patients: A Population-Based Cohort Study in Korea
Source: Front Psychiatry. 2021 May 26;12:668704. doi: 10.3389/fpsyt.2021.668704 (PMC8187563; doi:10.3389/fpsyt.2021.668704)
Supplement: Supplementary file 1 [file Table_1.DOCX]

**Association between antipsychotic treatment and neurological adverse events in pediatric patients: a population-based cohort study in Korea**

Soo Min Jeon, MS^1^*, Susan Park, PhD^1^*, Soonhak Kwon MD^2^, and Jin-Won Kwon, PhD^3^

* Soo Min Jeon and Susan Park contributed equally as co-first authors.

1. College of Pharmacy and Research Institute of Pharmaceutical Sciences, Kyungpook National University, Daegu, Republic of Korea
2. Department of Pediatric Neurology, Kyung-pook National University Children’s Hospital, and Kyungpook National University School of Medicine
3. BK21 FOUR Community-Based Intelligent Novel Drug Discovery Education Unit, College of Pharmacy and Research Institute of Pharmaceutical Sciences, Kyungpook National University, Daegu 41566, South Korea

**Table of contents**

| Supplementary Table 1: | ICD-10 code for movement disorders and seizures | p 3 |
| --- | --- | --- |
| Supplementary Table 2: | ATC codes for other psychotropic drugs | p 4 |
| Supplementary Table 3: | Antiparkinsonian drugs | p 5 |
| Supplementary Table 4: | ICD-10 code for record of mental health conditions | p 6 |
| Supplementary Table 5: | Risk of developing movement disorders or seizures according to the exposure status of antipsychotics during the antipsychotic exposure period (the duration of carry-over effect was assumed as 7 days) | p 7 |
| Supplementary Table 6: | Risk of developing movement disorders or seizures according to the cumulative duration of antipsychotic exposure period among the exposure period (the duration of carry-over effect was assumed as 7 days) | p 8 |
| Supplementary Table 7: | Risk of developing movement disorders or seizures according to antipsychotic polypharmacy during the antipsychotic exposure period (the duration of carry-over effect was assumed as 7 days) | p 9 |
| Supplementary Table 8: | Risk of developing movement disorders or seizures according to antipsychotic agent during the antipsychotic exposure period (the duration of carry-over effect was assumed as 7 days) | p 10 |
| Supplementary Table 9: | Risk of developing movement disorders or seizures according to the exposure status of antipsychotics (restricted follow-up for 1 year) | p 11 |
| Supplementary Table 10: | Risk of developing movement disorders or seizures according to the cumulative duration of antipsychotic exposure period among the exposure period (restricted follow-up for 1 year) | p 12 |
| Supplementary Table 11: | Risk of developing movement disorders or seizures according to antipsychotic polypharmacy during the antipsychotic exposure period (restricted follow-up for 1 year) | p 13 |
| Supplementary Table 12: | Risk of developing movement disorders or seizures according to antipsychotic agent during the antipsychotic exposure period (restricted follow-up for 1 year) | p 14 |
| Supplementary Table 13: | Summary statistics of antipsychotic average daily dose (mg/day)* in monotherapy and polypharmacy | p 15 |

**Table 1. ICD-10 code for movement disorders and seizures**

| **Diagnosis** | **ICD-10 code** |
| --- | --- |
| Movement disorders | G20, G21, G21.1, G21.2, G21.8, G21.9, G24, G24.0, G24.2, G25, G25.0, G25.1, G25.4, G25.6, and G25.9 |
| Seizures | R56.8 and G40 |
| ICD-10 code, International Classification of Diseases, 10th Edition | |

**Table 2. ATC codes for other psychotropic drugs**

| **Classification** | **ATC code** | **Agents in that classification** |
| --- | --- | --- |
| Antiepileptic drugs | N03A | Phenobarbital, Primidone, Phenytoin, Fosphenytoin, Ethosuximide, Clonazepam, Carbamazepine, Oxcarbazepine, Rufinamide, Valproic acid, Vigabatrin, Lamotrigine, Topiramate, Gabapentin, Levetiracetam, Zonisamide, Pregabalin, Stiripentol, Lacosamide |
| Anticholinergic drugs | N04A | Trihexyphenidyl, Biperiden, Procyclidine, Benztropine |
| Antianxiety drugs | N05B | Diazepam, Chlordiazepoxide, Lorazepam, Bromazepam, Clobazam, Alprazolam, Ethyl loflazepate, Etizolam, Clotiazepam, Tofisopam, Hydroxyzine, Buspirione |
| Antidepressants drugs | N06A | Fluoxetine, Citalopram, Paroxetine, Sertraline, Fluvoxamine, Escitalopram, Venlafaxine, Milnacipran, Duloxetine, Desvenlafaxine, Clomipramine, Amitriptyline, Doxepin, Amoxapine, Moclobemide, Trazodone, Mirtazapine, Bupropion, Tianeptine |
| Stimulants for ADHD | N06B | Methylphenidate |
| Nonstimulants for ADHD | N06B | Modafinil, Atomoxetine, Clonidine |
| ATC, Anatomical Therapeutic Chemical; ADHD, Attention Deficit Hyperactivity Disorder | | |

| **Table 3. Antiparkinsonian drugs** | | |
| --- | --- | --- |
| **Classification** | **ATC code** | **Agents in that classification** |
| Dopaminergic agents | [N04B](https://www.druginfo.co.kr/p/product-search/atc/?atcCode=N04BC02) | Pergolide, ropinirole, selegiline, levodopa and decarboxylase inhibitor, pramipexole, entacapone, rasagiline |
| ATC, Anatomical Therapeutic Chemical | | |

**Table 4. ICD-10 code for record of mental health conditions**

| **Psychiatric disorder** | **ICD-10 code** |
| --- | --- |
| Anxiety disorder | F40, F41 |
| Depression | F32, F33 |
| ADHD | F90 |
| Mental retardation | F70-F79 |
| Tic disorder | F95 |
| Bipolar disorder | F31 |
| Schizophrenia spectrum | F20-F29 |
| Autism spectrum disorder | F84 |
| ICD-10 code, International Classification of Diseases, 10th Edition; ADHD, Attention Deficit Hyperactivity Disorder | |

| **Table 5. Risk of developing movement disorders or seizures according to the exposure status of antipsychotics during the antipsychotic exposure period (the duration of carry-over effect was assumed as 7 days)** | | | | |
| --- | --- | --- | --- | --- |
|  | Cases | Person-Years | Incidence rate per 100 Person-Years | Adjusted HR (95% CI) ^a^ |
| ***Movement disorders*** |  |  |  |  |
| Nonexposure period | 434 | 20,002 | 2.17 | 1.00 (Reference) |
| Exposure period | 1,460 | 12,043 | 12.12 | 6.50 (5.74–7.35) |
|  | Cases | Person-Years | Incidence rate per 100 Person-Years | Adjusted HR (95% CI) ^b^ |
| ***Seizures*** |  |  |  |  |
| Nonexposure period | 411 | 19,856 | 2.07 | 1.00 (Reference) |
| Exposure period | 856 | 13,424 | 6.38 | 2.78 (2.41–3.22) |
| a. Adjusted for sex, age, insurance type, inpatient history, and psychiatric diagnosis. To consider the severity of psychiatric disorder related to seizure occurrence, covariates of inpatient history and other psychiatric medication use considered in a time-dependent manner were used in these models. Other psychiatric medication was included as antianxiety drugs, antidepressant drugs, stimulants for ADHD, nonstimulants for ADHD, antiepileptic drugs, and lithium.  b. Adjusted for sex, age, insurance type, inpatient history, and psychiatric diagnosis. To consider the severity of psychiatric disorder related to seizure occurrence, covariates of inpatient history and other psychiatric medication use considered in a time-dependent manner were used in these models. Other psychiatric medication was included as anticholinergic drugs, antianxiety drugs, antidepressant drugs, stimulants for ADHD, nonstimulants for ADHD, and lithium.  HR, Hazard Ratio; CI, Confidence Intervals; ADHD, Attention Deficit Hyperactivity Disorder | | | | |

| **Table 6. Risk of developing movement disorders or seizures according to the cumulative duration of antipsychotic exposure period among the exposure period (the duration of carry-over effect was assumed as 7 days)** | | | | |
| --- | --- | --- | --- | --- |
| Cumulative duration of antipsychotic exposure period (months) | Cases | Person-Years | Incidence rate per 100 Person-Years | Adjusted HR (95% CI) ^a^ |
| ***Movement disorders*** |  |  |  |  |
| ~1 | 279 | 271 | 102.83 | 13.76 (11.83–16.02) |
| 1< ≤2 | 173 | 414 | 41.77 | 6.00 (5.06–7.12) |
| 2< ≤3 | 111 | 410 | 27.05 | 3.91 (3.19–4.79) |
| 3< ≤4 | 85 | 438 | 19.41 | 2.85 (2.27–3.58) |
| 4< ≤5 | 55 | 396 | 13.87 | 2.01 (1.53–2.64) |
| 5< ≤6 | 45 | 370 | 12.17 | 1.79 (1.32–2.43) |
| 6≤ | 712 | 9,743 | 7.31 | 1.00 (Reference) |
| Cumulative duration of antipsychotic exposure period (months) | Cases | Person-Years | Incidence rate per 100 Person-Years | Adjusted HR (95% CI) ^b^ |
| ***Seizures*** |  |  |  |  |
| ~1 | 104 | 270 | 38.55 | 8.79 (7.01–11.02) |
| 1< ≤2 | 69 | 413 | 16.70 | 3.96 (3.05–5.14) |
| 2< ≤3 | 53 | 414 | 12.81 | 2.96 (2.21–3.96) |
| 3< ≤4 | 54 | 443 | 12.18 | 2.86 (2.14–3.82) |
| 4< ≤5 | 35 | 405 | 8.65 | 2.00 (1.42–2.83) |
| 5< ≤6 | 28 | 378 | 7.42 | 1.73 (1.18–2.55) |
| 6≤ | 513 | 11,102 | 4.62 | 1.00 (Reference) |
| a. Adjusted for sex, age, insurance type, inpatient history, and psychiatric diagnosis. To consider the severity of psychiatric disorder related to seizure occurrence, covariates of inpatient history and other psychiatric medication use considered in a time-dependent manner were used in these models. Other psychiatric medication was included was antianxiety drugs, antidepressant drugs, stimulants for ADHD, nonstimulants for ADHD, antiepileptic drugs, and lithium.  b. Adjusted for sex, age, insurance type, inpatient history, and psychiatric diagnosis. To consider the severity of psychiatric disorder related to seizure occurrence, covariates of inpatient history and other psychiatric medication use considered in a time-dependent manner were used in these models. Other psychiatric medication was included as anticholinergic drugs, antianxiety drugs, antidepressant drugs, stimulants for ADHD, nonstimulants for ADHD, and lithium.  HR, Hazard Ratio; CI, Confidence Intervals; ADHD, Attention Deficit Hyperactivity Disorder | | | | |

| **Table 7. Risk of developing movement disorders or seizures according to antipsychotic polypharmacy during the antipsychotics exposure period (the duration of carry-over effect was assumed as 7 days)** | | | | | |
| --- | --- | --- | --- | --- | --- |
|  | Cases | Person-Years | Incidence rate per 100 Person-Years | Adjusted HR ^a^(95% CI) | |
|  |  |  |  | Model 1 | Model 2 |
| ***Movement disorders*** |  |  |  |  |  |
| Monotherapy | 1,049 | 9,808 | 10.69 | 1.00 (Reference) | 1.00 (Reference) |
| Polypharmacy | 411 | 2,235 | 18.39 | 1.58 (1.38–1.81) | 1.34 (1.16–1.54) |
| Dose ^b^ |  |  |  |  |  |
| Low dose | 198 | 2,654 | 7.46 | - | 1.00 (Reference) |
| Moderate dose | 289 | 3,015 | 9.59 | - | 1.31 (1.09–1.59) |
| High dose | 457 | 3,257 | 14.03 | - | 1.80 (1.50–2.15) |
| Very high dose | 516 | 3,117 | 16.55 | - | 1.93 (1.60–2.34) |
|  | Cases | Person-Years | Incidence rate per 100 Person-Years | Adjusted HR ^c^(95% CI) | |
|  |  |  |  | Model 1 | Model 2 |
| ***Seizures*** |  |  |  |  |  |
| Monotherapy | 602 | 10,112 | 5.95 | 1.00 (Reference) | 1.00 (Reference) |
| Polypharmacy | 254 | 3,313 | 7.67 | 1.23 (1.02–1.47) | 1.09 (0.90–1.31) |
| Dose ^b^ |  |  |  |  |  |
| Low dose | 142 | 2,759 | 5.15 | - | 1.00 (Reference) |
| Moderate dose | 175 | 3,262 | 5.36 | - | 1.05 (0.83–1.31) |
| High dose | 241 | 3,665 | 6.58 | - | 1.25 (1.01–1.55) |
| Very high dose | 298 | 3,738 | 7.97 | - | 1.51 (1.21–1.89) |
| a. Adjusted for sex, age, insurance type, inpatient history, and psychiatric diagnosis. To consider the severity of psychiatric disorder related to seizure occurrence, covariates of inpatient history and other psychiatric medication use considered in a time-dependent manner were used in these models. Other psychiatric medication was included as antianxiety drugs, antidepressant drugs, stimulants for ADHD, nonstimulants for ADHD, antiepileptic drugs, and lithium.  b. Average daily dose was calculated as chlorpromazine equivalent dose and categorized into four groups according to time-varying age in each cohort. c. Adjusted for sex, age, insurance type, inpatient history, and psychiatric diagnosis. To consider the severity of psychiatric disorder related to seizure occurrence, covariates of inpatient history and other psychiatric medication use considered in a time-dependent manner were used in these models. Other psychiatric medication was included as anticholinergic drugs, antianxiety drugs, antidepressant drugs, stimulants for ADHD, nonstimulants for ADHD, and lithium.  HR, Hazard Ratio; CI, Confidence Intervals; ADHD, Attention Deficit Hyperactivity Disorder | | | | | |

| **Table 8. Risk of developing movement disorders or seizures according to antipsychotic agent during the antipsychotic exposure period (the duration of carry-over effect was assumed as 7 days)** | | | | |
| --- | --- | --- | --- | --- |
| Monotherapy ^a^ | Cases | Person-Years | Incidence rate per 100 Person-Years | Adjusted HR (95% CI) ^b^ |
| ***Movement disorders*** |  |  |  |  |
| Risperidone | 429 | 4,840 | 8.86 | 1.00 (Reference) |
| Aripiprazole | 422 | 3,647 | 11.57 | 0.85 (0.73–0.99) |
| Quetiapine | 38 | 459 | 8.29 | 0.52 (0.36–0.75) |
| Olanzapine | 34 | 157 | 21.60 | 0.91 (0.62–1.34) |
| Haloperidol | 53 | 248 | 21.36 | 2.23 (1.63–3.07) |
| Other antipsychotic agent | 73 | 457 | 15.96 | 0.97 (0.73–1.27) |
| Monotherapy ^a^ | Cases | Person-Years | Incidence rate per 100 Person-Years | Adjusted HR (95% CI) ^c^ |
| ***Seizures*** |  |  |  |  |
| Risperidone | 258 | 4,925 | 5.24 | 1.00 (Reference) |
| Aripiprazole | 210 | 3,830 | 5.48 | 0.99 (0.81–1.21) |
| Quetiapine | 58 | 396 | 14.63 | 1.79 (1.30–2.46) |
| Olanzapine | 22 | 172 | 12.81 | 1.57 (0.99–2.51) |
| Haloperidol | 15 | 309 | 4.86 | 0.90 (0.53–1.52) |
| Other antipsychotic agent | 39 | 479 | 8.14 | 1.13 (0.80–1.60) |
| a. Antipsychotic exposure period with polypharmacy and doses are not displayed in the table, but are included in the extended Cox regression model to allow for appropriate estimation of treatment effects. b. Adjusted for sex, age, insurance type, inpatient history, and psychiatric diagnosis. To consider the severity of psychiatric disorder related to seizure occurrence, covariates of inpatient history and other psychiatric medication use considered in a time-dependent manner were used in these models. Other psychiatric medication was included as antianxiety drugs, antidepressant drugs, stimulants for ADHD, nonstimulants for ADHD, antiepileptic drugs, and lithium.  c. Adjusted for sex, age, insurance type, inpatient history, and psychiatric diagnosis. To consider the severity of psychiatric disorder related to seizure occurrence, covariates of inpatient history and other psychiatric medication use considered in a time-dependent manner were used in these models. Other psychiatric medication was included as anticholinergic drugs, antianxiety drugs, antidepressant drugs, stimulants for ADHD, nonstimulants for ADHD, and lithium.  HR, Hazard Ratio; CI, Confidence Intervals; ADHD, Attention Deficit Hyperactivity Disorder | | | | |

| **Table 9. Risk of developing movement disorders or seizures according to the exposure status of antipsychotics (restricted follow-up for 1 year)** | | | | |
| --- | --- | --- | --- | --- |
|  | Cases | Person-Years | Incidence rate per 100 Person-Years | Adjusted HR (95% CI) ^a^ |
| ***Movement disorders*** |  |  |  |  |
| Nonexposure period | 141 | 4,458 | 3.16 | 1.00 (Reference) |
| Exposure period | 858 | 5,248 | 16.35 | 15.54 (12.66–19.09) |
|  | Cases | Person-Years | Incidence rate per 100 Person-Years | Adjusted HR (95% CI) ^b^ |
| ***Seizures*** |  |  |  |  |
| Nonexposure period | 137 | 4,462 | 3.07 | 1.00 (Reference) |
| Exposure period | 461 | 5,534 | 8.33 | 5.28 (4.20–) |
| a. Adjusted for sex, age, insurance type, inpatient history, and psychiatric diagnosis. To consider the severity of psychiatric disorder related to seizure occurrence, covariates of inpatient history and other psychiatric medication use considered in a time-dependent manner were used in these models. Other psychiatric medication was included as antianxiety drugs, antidepressant drugs, stimulants for ADHD, nonstimulants for ADHD, antiepileptic drugs, and lithium.  b. Adjusted for sex, age, insurance type, inpatient history, and psychiatric diagnosis. To consider the severity of psychiatric disorder related to seizure occurrence, covariates of inpatient history and other psychiatric medication use considered in a time-dependent manner were used in these models. Other psychiatric medication was included as anticholinergic drugs, antianxiety drugs, antidepressant drugs, stimulants for ADHD, nonstimulants for ADHD, and lithium.  HR, Hazard Ratio; CI, Confidence Intervals; ADHD, Attention Deficit Hyperactivity Disorder | | | | |

| **Table 10. Risk of developing movement disorders or seizures according to the cumulative duration of antipsychotic exposure period among the exposure period (restricted follow-up for 1 year)** | | | | |
| --- | --- | --- | --- | --- |
| Cumulative duration of antipsychotic exposure period (months) | Cases | Person-Years | Incidence rate per 100 Person-Years | Adjusted HR (95% CI) ^a^ |
| ***Movement disorders*** |  |  |  |  |
| ~1 | 279 | 271 | 102.83 | 34.59 (27.66–43.26) |
| 1< ≤2 | 173 | 414 | 41.77 | 14.59 (11.54–18.45) |
| 2< ≤3 | 111 | 410 | 27.05 | 8.39 (6.42–10.97) |
| 3< ≤4 | 85 | 438 | 19.41 | 6.45 (4.83–8.61) |
| 4< ≤5 | 55 | 396 | 13.87 | 5.04 (3.64–6.96) |
| 5< ≤6 | 45 | 370 | 12.17 | 3.18 (2.17–4.65) |
| 6≤ | 712 | 9,743 | 7.31 | 1.00 (Reference) |
| Cumulative duration of  Antipsychotic exposure period  (months) | Cases | Person-Years | Incidence rate per 100 Person-Years | Adjusted HR (95% CI) ^b^ |
| ***Seizures*** |  |  |  |  |
| ~1 | 104 | 270 | 38.55 | 22.80 (17.00–30.58) |
| 1< ≤2 | 69 | 413 | 16.70 | 9.98 (7.28–13.68) |
| 2< ≤3 | 53 | 414 | 12.81 | 8.26 (5.93–11.51) |
| 3< ≤4 | 54 | 443 | 12.18 | 7.03 (5.00–9.89) |
| 4< ≤5 | 35 | 405 | 8.65 | 4.04 (2.68–6.08) |
| 5< ≤6 | 28 | 378 | 7.42 | 4.16 (2.74–6.33) |
| 6≤ | 513 | 11,102 | 4.62 | 1.00 (Reference) |
| a. Adjusted for sex, age, insurance type, inpatient history, and psychiatric diagnosis. To consider the severity of psychiatric disorder related to seizure occurrence, covariates of inpatient history and other psychiatric medication use considered in a time-dependent manner were used in these models. Other psychiatric medication was included as antianxiety drugs, antidepressant drugs, stimulants for ADHD, nonstimulants for ADHD, antiepileptic drugs, and lithium.  b. Adjusted for sex, age, insurance type, inpatient history, and psychiatric diagnosis. To consider the severity of psychiatric disorder related to seizure occurrence, covariates of inpatient history and other psychiatric medication use considered in a time-dependent manner were used in these models. Other psychiatric medication was included as anticholinergic drugs, antianxiety drugs, antidepressant drugs, stimulants for ADHD, nonstimulants for ADHD, and lithium.  HR, Hazard Ratio; CI, Confidence Intervals; ADHD, Attention Deficit Hyperactivity Disorder | | | | |

| **Table 11. Risk of developing movement disorders or seizures according to antipsychotic polypharmacy during the antipsychotic exposure period (restricted follow-up for 1 year)** | | | | | |
| --- | --- | --- | --- | --- | --- |
|  | Cases | Person-Years | Incidence rate per 100 Person-Years | Adjusted HR ^a^(95% CI) | |
|  |  |  |  | Model 1 | Model 2 |
| ***Movement disorders*** |  |  |  |  |  |
| Monotherapy | 638 | 4,666 | 13.67 | 1.00 (Reference) | 1.00 (Reference) |
| Polypharmacy | 220 | 582 | 37.81 | 1.92 (1.60–2.30) | 1.70 (1.39–2.08) |
| Dose ^b^ |  |  |  |  |  |
| Low dose | 132 | 1,042 | 12.67 | - | 1.00 (Reference) |
| Moderate dose | 175 | 1,467 | 11.93 | - | 1.01 (0.79–1.29) |
| High dose | 257 | 1,517 | 16.94 | - | 1.25 (0.99–1.57) |
| Very high dose | 294 | 1,222 | 24.06 | - | 1.38 (1.08–1.78) |
|  | Cases | Person-Years | Incidence rate per 100 Person-Years | Adjusted HR ^c^(95% CI) | |
|  |  |  |  | Model 1 | Model 2 |
| ***Seizures*** |  |  |  |  |  |
| Monotherapy | 360 | 4,765 | 7.55 | 1.00 (Reference) | 1.00 (Reference) |
| Polypharmacy | 101 | 769 | 13.13 | 1.40 (1.07–1.82) | 1.35 (1.02–1.79) |
| Dose ^b^ |  |  |  |  |  |
| Low dose | 90 | 1,079 | 8.34 | - | 1.00 (Reference) |
| Moderate dose | 108 | 1,518 | 7.11 | - | 0.90 (0.68–1.21) |
| High dose | 127 | 1,590 | 7.99 | - | 0.92 (0.70–1.22) |
| Very high dose | 136 | 1,347 | 10.09 | - | 1.03 (0.75–1.42) |
| a. Adjusted for sex, age, insurance type, inpatient history, and psychiatric diagnosis. To consider the severity of psychiatric disorder related to seizure occurrence, covariates of inpatient history and other psychiatric medication use considered in a time-dependent manner were used in these models. Other psychiatric medication was included as antianxiety drugs, antidepressant drugs, stimulants for ADHD, nonstimulants for ADHD, antiepileptic drugs, and lithium.  b. Average daily dose was calculated as chlorpromazine equivalent dose and categorized into four groups according to time-varying age in each cohort. c. Adjusted for sex, age, insurance type, inpatient history, and psychiatric diagnosis. To consider the severity of psychiatric disorder related to seizure occurrence, covariates of inpatient history and other psychiatric medication use considered in a time-dependent manner were used in these models. Other psychiatric medication was included as anticholinergic drugs, antianxiety drugs, antidepressant drugs, stimulants for ADHD, nonstimulants for ADHD, and lithium.  HR, Hazard Ratio; CI, Confidence Intervals; ADHD, Attention Deficit Hyperactivity Disorder | | | | | |

| **Table 12. Risk of developing movement disorders or seizures according to antipsychotic agent during the antipsychotic exposure period (restricted follow-up for 1 year)** | | | | |
| --- | --- | --- | --- | --- |
|  | Cases | Person-Years | Incidence rate per 100 Person-Years | Adjusted HR (95% CI) ^a^ |
| Movement disorders |  |  |  |  |
| Risperidone | 249 | 2,337 | 10.66 | 1.00 (Reference) |
| Aripiprazole | 268 | 1,603 | 16.71 | 0.89 (0.73–1.09) |
| Quetiapine | 23 | 234 | 9.85 | 0.48 (0.30–0.75) |
| Olanzapine | 21 | 87 | 24.14 | 0.79 (0.48–1.30) |
| Haloperidol | 32 | 131 | 24.35 | 2.32 (1.54–3.50) |
| Other antipsychotic agent | 45 | 274 | 16.43 | 0.76 (0.53–1.08) |
|  | Cases | Person-Years | Incidence rate per 100 Person-Years | Adjusted HR (95% CI) ^b^ |
| Seizures |  |  |  |  |
| Risperidone | 147 | 2,377 | 6.19 | 1.00 (Reference) |
| Aripiprazole | 123 | 1,650 | 7.46 | 1.11 (0.84–1.47) |
| Quetiapine | 45 | 223 | 20.16 | 2.09 (1.43–3.07) |
| Olanzapine | 13 | 92 | 14.12 | 1.62 (0.88–2.99) |
| Haloperidol | 8 | 141 | 5.68 | 0.85 (0.41–1.77) |
| Other antipsychotic agent | 24 | 283 | 8.47 | 0.96 (0.60–1.52) |
| a. Antipsychotic exposure period with polypharmacy and doses are not displayed in the table, but are included in the extended Cox regression model to allow for appropriate estimation of treatment effects. b. Adjusted for sex, age, insurance type, inpatient history, and psychiatric diagnosis. To consider the severity of psychiatric disorder related to seizure occurrence, covariates of inpatient history and other psychiatric medication use considered in a time-dependent manner were used in these models. Other psychiatric medication was included as antianxiety drugs, antidepressant drugs, stimulants for ADHD, nonstimulants for ADHD, antiepileptic drugs, and lithium.  c. Adjusted for sex, age, insurance type, inpatient history, and psychiatric diagnosis. To consider the severity of psychiatric disorder related to seizure occurrence, covariates of inpatient history and other psychiatric medication use considered in a time-dependent manner were used in these models. Other psychiatric medication was included as anticholinergic drugs, antianxiety drugs, antidepressant drugs, stimulants for ADHD, nonstimulants for ADHD, and lithium.  HR, Hazard Ratio; CI, Confidence Intervals; ADHD, Attention Deficit Hyperactivity Disorder | | | | |

| **Table 13. Summary statistics of antipsychotic average daily dose (mg/day)* in monotherapy and polypharmacy** | | | | | |
| --- | --- | --- | --- | --- | --- |
|  |  | **Movement disorders** | | **Seizures** | |
|  |  | **Monotherapy** | **Polypharmacy** | **Monotherapy** | **Polypharmacy** |
| All patients | Mean | 110.50 | 656.79 | 110.75 | 655.22 |
|  | SD | 164.67 | 1,750.37 | 163.91 | 1,705.50 |
| 2–6 | Mean | 61.39 | 162.96 | 62.21 | 169.23 |
|  | SD | 74.58 | 142.71 | 74.75 | 150.04 |
| 7–12 | Mean | 79.14 | 352.33 | 79.24 | 356.18 |
|  | SD | 89.06 | 559.09 | 89.11 | 561.72 |
| 13–18 | Mean | 125.65 | 728.70 | 125.95 | 725.25 |
|  | SD | 187.83 | 1913.69 | 186.86 | 1862.64 |
| * SD, Standard Deviation a. Average daily dose was calculated based on chlorpromazine equivalents | | | | | |
